# Correlation between systemic inflammatory response index and thyroid function: 2009-2012 NHANES results

**DOI:** 10.3389/fendo.2023.1305386

**Published:** 2024-01-22

**Authors:** Yuze Zhai, Benjun Wang, Weiwei Han, Bianfang Yu, Jichen Ci, Fan An

**Affiliations:** ^1^ First Clinical Medical College, Shandong University of Traditional Chinese Medicine, Jinan, Shandong, China; ^2^ Department of Anal Surgery, Affiliated Hospital of Shandong University of Traditional Chinese Medicine, Jinan, Shandong, China; ^3^ College of Traditional Chinese Medicine, Shandong University of Traditional Chinese Medicine, Jinan, Shandong, China

**Keywords:** thyroid hormone, systemic inflammatory response index, thyroid function, positive correlation, national health and nutrition examination survey

## Abstract

**Aims:**

This study investigates the relationship between the Systemic Inflammatory Response Index (SIRI) and thyroid function.

**Methods:**

Utilizing data from the National Health and Nutrition Examination Survey (NHANES) 2009-2012, we excluded participants lacking SIRI or thyroid function data, those under 20 years, and pregnant individuals. SIRI was determined using blood samples. We conducted weighted multivariate regression and subgroup analyses to discern the independent relationship between SIRI and thyroid function.

**Results:**

The study included 1,641 subjects, with an average age of 47.26±16.77 years, including 48.65% males and 51.35% females. The population was divided into three SIRI-based groups (Q1-Q3). Q3, compared to Q1, exhibited higher age-at-onset, greater male prevalence, and increased levels of FT3, FT4, TT4, leukocytes, and triglycerides. This group also showed a higher incidence of diabetes, hypertension, and smoking. Notably, Q1 had lower LDL and HDL levels. SIRI maintained a positive association with FT4 (*β* = 0.01, 95% CI = 0.00-0.03, P for trend = 0.0071), TT4 (*β* = 0.20, 95% CI = 0.10, 0.31, *P* for trend=0.0001), and TPOAb (*β* = 8.0, 95% CI = 1.77-14.30, *P* for trend = 0.0120), indicating that each quartile increase in SIRI corresponded to a 0.01 ng/dL increase in FT4, a 0.2 g/dL increase in TT4, and an 8.03 IU/mL rise in TPOAb. The subgroup analysis suggested the SIRI-thyroid function correlation was influenced by hypertension.

**Conclusion:**

Inflammation may impact the development and progression of thyroid function disorders. Proactive anti-inflammatory treatment might mitigate thyroid abnormalities.

## Introduction

The thyroid gland, the largest endocrine gland in adults, plays a pivotal role in regulating physiological activities (1) and is one of the most important components of the human body. Thyroid hormones (TH), primarily triiodothyronine (T3) and tetraiodothyronine (T4) are crucial for systemic metabolism, neurodevelopment, and energy metabolism, influencing protein, carbohydrate, and lipid metabolism. Recent studies indicate a rising prevalence of thyroid disorders, now second only to diabetes mellitus as a global metabolic disease. These disorders often lead to developmental and cognitive impairments, osteoporosis, and insomnia, significantly impacting patients' physical and mental health (2).

TH comprises both T3 and significant quantities of T4. The active free thyroid hormone in the body consists of free triiodothyronine (Free T3, FT3) and free thyroxine (Free T4, FT4), but when FT3 and FT4 are depleted, total triiodothyronine (Total T3, TT3) and total thyroxine (Total T4, TT4) are converted to FT3 and FT4 in the body, which continues to play the role of TH. Although TH is synthesized in the follicular epithelial cells of the thyroid gland, its synthesis, and secretion depend on the stimulation of thyroid-stimulating hormone (TSH). Thyroid peroxidase antibodies (TPOAb) are found in the microsomes of thyroid cells and are a key enzyme in the synthesis of TH. They work in concert with Thyreoglobulin to iodide L-tyrosine into TH, which is a common autoantibody in the serum of patients with autoimmune thyroid disease. It is seen in autoimmune thyroid diseases such as Hashimoto's thyroiditis (HT) (3).

Inflammation response is a defense response of the organism itself when tissues are damaged or infected by toxins or bacteria, injured by heat or other causes, they go into a normal state of self-protection and damage repair. The System Inflammation Response Index (SIRI) is a new systemic inflammation measure created in 2016 by Qi et al. (4). SIRI (1000cell/uL) = N×M/L, as the calculation method, where N, M, and L are the pretreatment peripheral neutrophil, monocyte, and lymphocyte counts, respectively. Previous studies have concluded that SIRI can be used to differentiate the immune-inflammatory response of the body's three different pathways, and to reflect the body's immune response and inflammation in a more comprehensive way. Subsequently, this index was used to measure the recurrence rate and survival rate after cancer surgery. In addition, the index has been used to measure the degree of brain damage in aneurysmal arachnoiditis (5) hemorrhage, and the development and prognosis of the Corona Virus Disease 2019 (COVID-19).

The relationship between inflammation and thyroid function, though correlated, remains poorly understood. Therefore, our study aims to elucidate the impact of inflammation on thyroid disease development by examining the correlation between SIRI and thyroid function in participants of the National Health and Nutrition Examination Survey (NHANES). This could pave the way for novel strategies in the primary prevention of thyroid diseases. We hypothesized that elevated SIRI is associated with a higher risk of abnormal thyroid function.

## Materials and methods

### Study design and population

**Figure 1:**
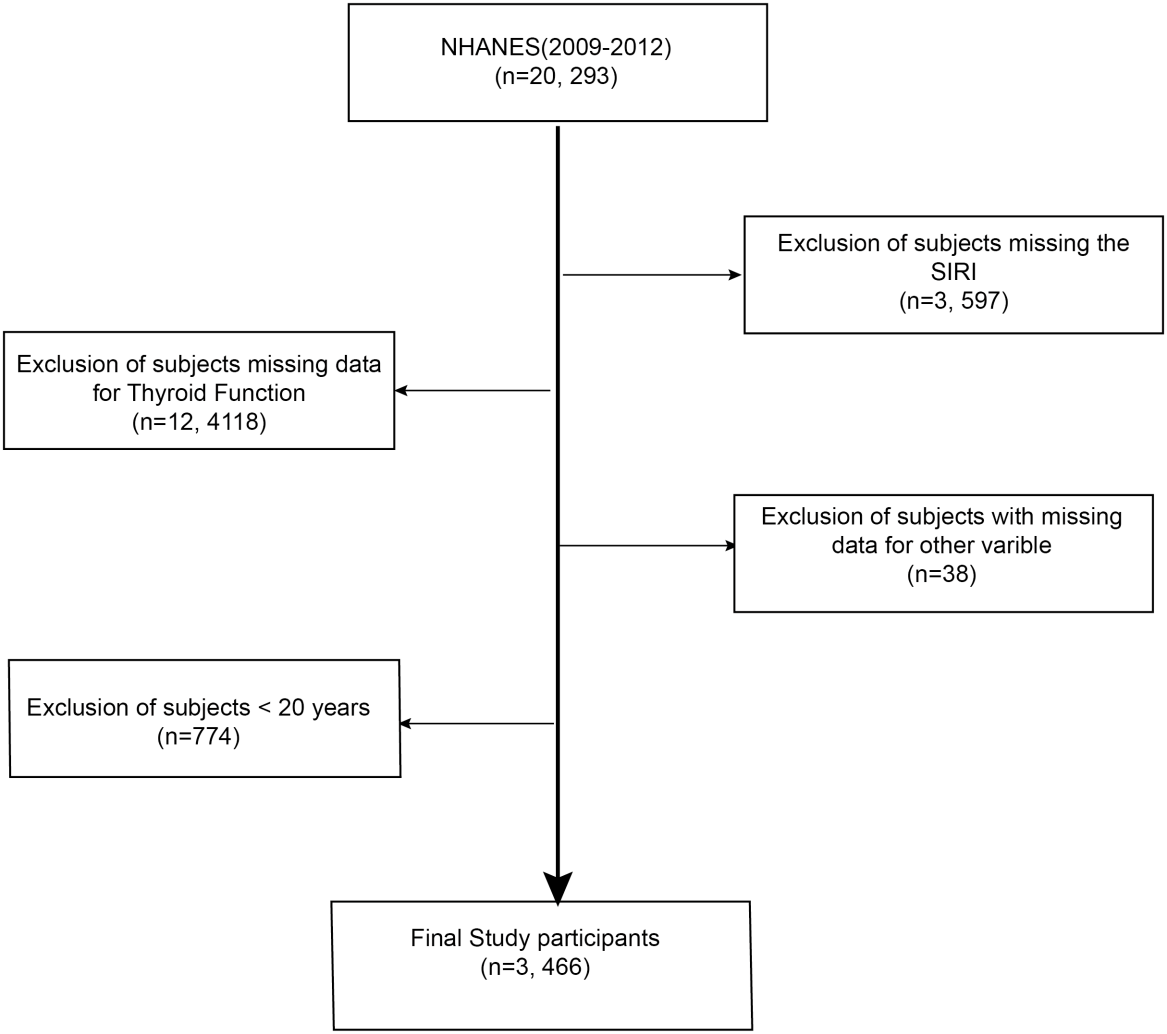
Selection process for the study sample based on the 2009-2012 National Health and Nutrition Examination Survey (NHANES).

Data for this study were sourced from the National Health and Nutrition Examination Survey (NHANES), a biennial cross-sectional study conducted by the Centers for Disease Control and Prevention (U.S.) to assess the health and nutritional status of American citizens. The NHANES study protocol adhered to the Declaration of Helsinki principles and received approval from the Institutional Review Board, with all participants providing written informed consent. Data were accessed from NHANES's public domain (www.example.com; https://www.cdc.gov/nchs/nhanes/, accessed on 12 August 2023), and **Figure 1** depicts the participant selection process.

This research utilized data from the 2009-2012 NHANES survey cycle, which encompassed comprehensive data on thyroid function, the System Inflammation Response Index (SIRI), and demographic information. The final study cohort, after excluding individuals with incomplete SIRI or thyroid function data, those under 20 years of age, and pregnant women, comprised 1,641 participants. All participants consented to the study, and the Ethical Review Board of the National Centre for Health Statistics authorized the research.

### Measurement of peripheral blood cell counts

All blood specimens (neutrophil count, monocyte count, and lymphocyte count) were collected at Mobile Examination Centers (MECs). The procedures for quality assurance and control of these specimens adhered to the guidelines outlined in the NHANES Laboratory Procedures Manual, which can be accessed at https://wwwn.cdc.gov/nchs/data/nhanes/2009-2010/labmethods/CBC_F_met_HE.pdf.

The SIRI was defined as follows: SIRI (1000cell/uL) = N×M/L, where N, M, and L are the pretreatment peripheral neutrophil, monocyte, and lymphocyte counts, respectively (4).

### Measurement of thyroid outcomes

This study involved the analysis of serum samples for thyroid function indicators including FT3, FT4, TSH, TPOAb, TT3, and TT4. The methods for serum specimen collection and processing are elaborately described in the National Health and Nutrition Examination Survey (NHANES) Laboratory Procedures Manual (available at: wwwn.cdc.gov/nchs/nhanes/2007-2008/THYROD_E.htm).

### The covariates

The study accounted for various covariates potentially influencing thyroid function: age, gender (male, female), educational attainment (below high school, high school or equivalent, college graduate or above), marital status (married or not), and race/ethnicity (Mexican American, non-Hispanic White, non-Hispanic Black, and others). Diabetes mellitus was defined as any self-reported diagnosis of diabetes mellitus or self-reported use of insulin or antidiabetic medication or fasting blood glucose greater than 7.0 mmol/L or glycated hemoglobin greater than 6%. Hypertension was defined as any self-reported diagnosis of hypertension or self-reported use of blood pressure-lowering medication or SBP ≥ 140 mm Hg or DBP ≥ 90 mm Hg. Body Mass Index (BMI) was determined from the measured weight and height. Serum biochemistry analyses included total High-Density Lipoprotein (HDL) cholesterol, total Low-Density Lipoprotein (LDL) cholesterol, and triglycerides.

### Statistical methods

Statistical analyses in this study were conducted using R packages (The R Foundation: http://www.r-project.org;version3.4.3) and Empower (R) (www.empowerstats.com, X&Y Solutions, Inc., Boston, Massachusetts). Given the complex multi-stage sampling design of the National Health and Nutrition Examination Survey (NHANES), this study utilized MEC exam weights (WTMEC4YR, WTMEC2YR) for analysis. In this study, categorical variables were expressed as weighted percentages, while continuous variables were expressed as weighted means and standard deviations. Differences in characteristics between groups were tested by chi-square test (for categorical variables) and one-way ANOVA (for continuous variables). All statistical tests were two-sided, and a *P* value< 0.05 was considered statistically significant.

Furthermore, the correlation between the SIRI and thyroid function was analyzed using weighted multivariate logistic regression models. SIRI was treated as a categorical variable in quartiles. Three models were employed: Model 1, without covariate adjustment; Model 2, adjusting for gender, age, and race; and Model 3, further adjusting for education level and marital status. To visually depict the relationship between SIRI and thyroid function, smoothed curve fitting was used. In these plots, the central line represents the effect size, while the surrounding area indicates the 95% CI. *P* value <0.05 was deemed statistically significant.

## Results

### Baseline characteristics of the study population

The study's baseline characteristics are detailed in **Table 1**, which outlines the demographic and laboratory parameters of the participants. The study encompassed 3,466 individuals, with an average age of 47.26 ± 16.77 years, comprising 48.65% males and 51.35% females. Participants were stratified into three groups according to the tertiles of the Systemic Immune-Inflammation Index (SIRI): the first tertile group (Q1, N=1,130), the second tertile group (Q2, N=1,150), and the third tertile group (Q3, N=1,185). Notably, the Q3 group, compared to Q1, demonstrated a higher mean age, a greater male proportion, and elevated levels of FT3, FT4, TT4,WBC, and triglycerides. This group also showed a higher prevalence of diabetes, hypertension, and smoking. In contrast, lower levels of LDL and HDL were observed in the Q1 group (all *P* < 0.05).

**Table 1:** Baseline characteristics of participants in the 2009–2012 continuous NHANES, weighted.

Characteristics	Systemic inflammatory response index (SIRI (1000 cells /UL)) ^a^			*P*-Value ^b^
	Q1 N=1,130	Q2 N=1,150	Q3 N=1,185	
**Age (years)**	45.60 ± 15.68^c^	46.55 ± 16.46	49.34 ± 17.72	<0.0001
**Gender (%)**				<0.0001
Male	41.94^d^	47.14	55.75	
Female	58.06	52.86	44.25	
**Race (%)**				<0.0001
Mexican American	9.33	7.52	6.87	
Other Hispanic	6.43	5.63	5.92	
Non-Hispanic White	56.36	70.53	76.01	
Non-Hispanic Black	18.28	8.80	5.44	
Other Race - Including Multi-Racial	9.61	7.51	5.77	
**Education Level (%)**				0.2895
Under high school	17.01	16.29	18.86	
High school or equivalent	21.22	20.98	22.48	
College graduate or above	61.77	62.73	58.66	
**Marital Status (%)**				0.0723
Yes	52.10	56.91	54.22	
No	47.90	43.09	45.78	
**Diabetes (%)**				0.0146
Yes	33.25	29.04	34.28	
No	66.75	70.96	65.72	
**Hypertension (%)**				<0.0001
Yes	31.29	30.87	41.78	
No	68.71	69.13	58.22	
**Smoking status (%)**				<0.0001
Current smoker	13.57	14.90	21.17	
Former smoker	4.21	2.71	3.66	
Never	82.21	82.39	75.18	
**BMI (kg/m^2^)**	28.09 ± 6.61	28.43 ± 6.22	29.34 ± 7.13	<0.0001
**FT3 (pg/mL)**	3.14 ± 0.38	3.18 ± 0.38	3.17 ± 0.39	0.0209
**FT4 (ng/dL)**	0.81 ± 0.15	0.82 ± 0.17	0.83 ± 0.16	0.0455
**TSH(uIU/mL)**	2.10 ± 5.29	1.87 ± 1.62	2.07 ± 3.64	0.2707
**TPOAb(IU/mL)**	17.03 ± 78.29	25.42 ± 101.26	24.12 ± 99.45	0.0847
**TT3 (ng/dL)**	113.08 ± 24.50	115.2±23.88	114.36 ±22.81	0.1049
**TT4 (µg/dL)**	7.82 ± 1.56	7.96 ± 1.72	8.02 ± 1.56	0.0105
**WBC (1000cells/uL)**	5.81 ± 1.53	6.76 ± 1.57	8.31 ± 2.36	<0.0001
**Triglyceride (mg/dL)**	124.21 ± 89.98	126.92±109.34	140.68±130.72	0.0348
**LDL-cholesterol (mg/dL)**	119.66 ± 36.53	113.68±31.63	115.44 ± 35.26	0.0110
**Direct HDL-Cholesterol (mg/dL)**	54.73 ± 16.50	53.67 ± 16.51	51.09 ± 15.29	<0.0001

Values in bold indicate significance at P < 0.05.

BMI, body mass index;

^a^Systemic inflammatory response index(SIRI) was divided into three groups, Q1 and Q3 are the lowest and highest quintile groups, respectively.

^b^ANOVA and chi-square tests are used to assess the significance of continuous and categorical variables, respectively.

^c^Continuous values are presented as mean and standard error.

^d^Categorical values are presented as %.

TT3, Free Triiodothyronine;

FT4, Free Thyroxine;

TSH, Thyroid stimulating hormone;

TPOAb, Thyroid peroxidase antibodies;

TT3, Total Triiodothyronine;

TT4, Total Thyroxine;

WBC, White blood cell count.

### Univariate analysis of thyroid function

**Table 2** presents the results of the univariate analyses. In our study population, thyroid function showed no association with race. However, a significant positive correlation was observed between and marital status (*β* = 0.08, 95% CI = 0.05, 0.10), as well as WBC (*β* = 0.01, 95% CI = 0.00, 0.01), negatively correlated with age (*β* = -0.01, 95% CI = 0.01, 0.00) and gender (*β* = -0.09, 95% CI = -0.21, -0.16).

**Table 2:** Univariate analysis of thyroid function, weighted.

	FT3 (pg/mL)	FT4 (ng/dL)	TSH(uIU/mL)	TPOAb(IU/mL)	TT3 (ng/dL)	TT4 (µg/dL)
Age, years	-0.01 (-0.01,-0.0) <0.0001	0.00 (0.00, 0.00) <0.0001	0.01 (0.00, 0.02) 0.0084	0.13 (-0.06, 0.31) 0.1871	-0.38 (-0.42,-0.3) <0.0001	0.01 (0.00, 0.01) <0.0001
Gender,n%						
Male	Ref.	Ref.	Ref.	Ref.	Ref.	Ref.
Female	-0.19 (-0.21,-0.16) <0.0001	0.01 (-0.01,0.02) 0.3276	0.33 (0.08, 0.58) 0.0102	18.12 (11.83,24.40) <0.0001	-2.07 (-3.65,-0.49) 0.0103	0.49 (0.39, 0.60) <0.0001
Race, n%						
Mexican American	Ref.	Ref.	Ref.	Ref.	Ref.	Ref.
Other Hispanic	0.01 (-0.06, 0.08) 0.7978	0.02 (-0.01,0.05) 0.2409	-0.00 (-0.68,0.67) 0.9977	-4.14 (-21.36,13.07) 0.6372	0.84 (-3.44,5.12) 0.6992	-0.05 (-0.34, 0.25) 0.7606
Non-Hispanic White	-0.13 (-0.18,-0.08) <0.0001	0.01 (-0.01,0.03) 0.2838	0.15 (-0.31,0.62) 0.5193	3.94 (-8.03, 15.91) 0.5189	-4.99 (-7.96,-2.01) 0.0010	-0.41 (-0.61,-0.21) <0.0001
Other Hispanic	-0.09 (-0.15,-0.03) 0.0022	0.01 (-0.02,0.03) 0.6731	-0.50 (-1.09, 0.09) 0.0939	-6.99 (-21.96, 7.98) 0.3604	-4.54 (-8.27,-0.82) 0.0169	-0.13 (-0.39, 0.12) 0.3052
Other Race	-0.09 (-0.15,-0.02) 0.0094	0.05 (0.03, 0.08) 0.0001	-0.38 (-1.01,0.26) 0.2455	-0.33 (-16.46,15.80) 0.9681	-4.07 (-8.09,-0.05) 0.0473	-0.01 (-0.29, 0.26) 0.9350
Marital Status, n%						
Yes	Ref.	Ref.	Ref.	Ref.	Ref.	Ref.
No	0.08 (0.05, 0.10) <0.0001	0.00 (-0.01,0.01) 0.4480	0.04 (-0.21, 0.29) 0.7701	0.24 (-6.10, 6.58) 0.9405	3.21 (1.63, 4.79) <0.0001	0.06 (-0.05, 0.16) 0.3175
Education Level(%)						
Under high school	Ref.	Ref.	Ref.	Ref.	Ref.	Ref.
High school or equivalent	-0.00 (-0.04, 0.04) 0.8685	-0.02 (-0.04,-0.0) 0.0164	0.14 (-0.26, 0.54) 0.4972	-2.00 (-12.16, 8.16) 0.6997	0.06 (-2.48,2.59) 0.9655	-0.45 (-0.62,-0.27) <0.0001
College graduate or above	-0.06 (-0.09,-0.03) 0.0007	-0.02 (-0.03,-0.0) 0.0415	0.22 (-0.11, 0.56) 0.1946	6.82 (-1.74, 15.38) 0.1185	-4.33 (-6.46,-2.19) <0.0001	-0.47 (-0.62,-0.33) <0.0001
WBC (1000cells/uL)	0.01 (0.00, 0.01) 0.0069	0.00 (-0.00,0.00) 0.8978	0.01 (-0.05, 0.06) 0.8463	-0.57 (-2.05, 0.90) 0.4450	0.91 (0.54, 1.27) <0.0001	0.08 (0.06, 0.11) <0.0001
SIRI (1000cells/uL)	-0.01 (-0.03, 0.01) 0.2162	0.01 (0.01, 0.02) <0.0001	0.01 (-0.15, 0.17) 0.9175	2.68 (-1.31, 6.67) 0.1883	-0.91 (-1.91, 0.09) 0.0743	0.10 (0.03, 0.17) 0.0046

SIRI, Systemic inflammatory response index;

TT3, Free Triiodothyronine;

FT4, Free Thyroxine;

TSH, Thyroid stimulating hormone;

TPOAb, Thyroid peroxidase antibodies;

TT3, Total Triiodothyronine;

TT4, Total Thyroxine;

CI, confidence interval.

Regarding FT4, a significant positive correlation was noted with age (*β* = 0.00, 95% CI = 0.00, 0.00) and the SIRI (*β* = 0.01, 95% CI = 0.01, 0.02), while a negative correlation was observed with education level (*β* = -0.02, 95% CI = -0.03, -0.0). TSH and TPOAb both demonstrated a positive correlation with gender (TSH: *β* = 0.33, 95% CI = 0.08, 0.58; TPOAb: *β* = 18.12, 95% CI = 11.83, 24.40), with TSH also positively correlating with age (*β* = 0.01, 95% CI = 0.00, 0.02). TT3 showed a negative correlation with both age (*β* = -0.38, 95% CI = -0.42, 0.34) and gender (*β* = -2.07, 95% CI = -3.65, -0.49), while positively correlating with marital status (*β* = 3.21, 95% CI = 1.63, 4.79) and WBC (*β* = 0.91, 95% CI = 0.54, 1.27). Finally, TT4 exhibited a positive association with age (*β* = 0.01, 95% CI = 0.00, 0.01), gender (*β* = 0.49, 95% CI=0.39, 0.60), WBC (*β* = 0.08, 95% CI = 0.06, 0.11), and SIRI (*β* = 0.10, 95% CI = 0.03,0.17), but a negative correlation with education level (*β* = -0.47, 95% CI = -0.62, -0.33).

### Relationship between SIRI and thyroid function

**Table 3** presents the results of a weighted multivariate linear regression analysis, examining the relationship between the SIRI and thyroid function indicators. The results of the study showed that SIRI was significantly and positively correlated with FT4 and TT4 in thyroid function (FT4: Model 1, *β* = 0.01, 95% CI = 0.01, 0.02; Model 2, *β* = 0.01, 95% CI = 0.01, 0.02; Model 3, *β* = 0.01, 95% CI = 0.01, 0.02; TT4: Model 1, *β* =0.1, 95% CI = 0.03, 0.17; Model 2, *β* =0.16, 95% CI = 0.09, 0.23; Model 3, *β* =0.15, 95% CI = 0.08, 0.22), but there was no statistically significant relationship with FT3, TSH, TgAb, and TT3. Subsequently, we transformed SIRI into quartile categorical variables, and in the fully adjusted model (Model 3) we found that SIRI was associated with FT4 (*β* = 0.01, 95% CI = 0.00, 0.03, *P* for trend = 0.0071), TT4 (*β* = 0.20, 95% CI = 0.10, 0.31, *P* for trend = 0.0001), and TPOAb (*β* = 8.03, 95% CI = 1.77, 14.30, *P* for trend = 0.012) remained positively correlated with each other. This suggests that an increase in SIRI per quartile was associated with an increase in FT4 of 0.01ng/dL, an increase in TT4 of 0.2µg/dL, and an elevation in TPOAb of 8.03IU/mL. No statistically significant differences were found among SIRI and FT3, TSH, and TT3 even in the SIRI quartiles.

**Table 3:** Association between SIRI and thyroid function among U.S. adults in NHANES from 2009-2012, weighted.

SIRI Tertile	FT3 (pg/mL)	FT4(ng/dL)	TSH(uIU/mL)	TPOAb(IU/mL)	TT3 (ng/dL)	TT4(µg/dL)
*β* (95% CI), *P* Value^a^						
Model 1						
Continuous	-0.01 (-0.03, 0.01) 0.2162	0.01 (0.01, 0.02) <0.0001	0.01 (-0.15, 0.17) 0.9175	2.68 (-1.31, 6.67) 0.1883	-0.91 (-1.91, 0.09) 0.0743	0.10 (0.03, 0.17) 0.0046
Tertile 1	Ref.	Ref.	Ref.	Ref.	Ref.	Ref.
Tertile 2	0.03 (-0.01, 0.06) 0.1735	0.01 (-0.01, 0.03) 0.2113	-0.29 (-0.65, 0.07) 0.1148	6.95 (-2.22, 16.11) 0.1377	-0.27 (-2.56, 2.03) 0.8206	0.10 (-0.06, 0.26) 0.2060
Tertile 3	0.05 (0.01, 0.09) 0.0070	0.02 (0.00, 0.03) 0.0251	-0.25 (-0.61, 0.10) 0.1649	10.22 (1.21, 19.23) 0.0263	3.50 (1.25, 5.75) 0.0023	0.26 (0.11, 0.41) 0.0009
Tertile 4	0.02 (-0.02, 0.06) 0.3023	0.02 (0.01, 0.04) 0.0033	-0.16 (-0.51, 0.20) 0.3885	10.52 (1.49, 19.56) 0.0225	0.04 (-2.22, 2.30) 0.9734	0.17 (0.02, 0.33) 0.0266
*p* for trend	0.01 (-0.02, 0.03) 0.4625	0.02 (0.01, 0.03) 0.0036	-0.05 (-0.29, 0.19) 0.6892	6.47 (0.37, 12.57) 0.0377	0.20 (-1.32, 1.73) 0.7943	0.11 (0.01, 0.22) 0.0321
Model 2						
Continuous	-0.01 (-0.02, 0.01) 0.4066	0.01 (0.01, 0.02) 0.0002	-0.03 (-0.19, 0.14) 0.7500	3.55 (-0.53, 7.62) 0.0879	-0.34 (-1.33, 0.65) 0.5005	0.16 (0.09, 0.23) <0.0001
Tertile 1	Ref.	Ref.	Ref.	Ref.	Ref.	Ref.
Tertile 2	0.03 (-0.00,0.06) 0.0757	0.01 (-0.00, 0.03) 0.1818	-0.36 (-0.72, 0.00) 0.0521	6.23 (-2.95, 15.40) 0.1835	-0.16 (-2.37, 2.06) 0.8908	0.14 (-0.02, 0.29) 0.0838
Tertile 3	0.07 (0.03, 0.10) <0.0001	0.02 (0.00, 0.03) 0.0278	-0.36 (-0.72, -0.00) 0.0483	9.68 (0.58, 18.77) 0.0371	4.29 (2.09, 6.48) 0.0001	0.34 (0.19, 0.49) <0.0001
Tertile 4	0.03 (-0.00, 0.06) 0.0894	0.02 (0.01, 0.04) 0.0039	-0.27 (-0.63, 0.10) 0.1506	12.15 (2.90, 21.41) 0.0101	1.26 (-0.98, 3.49) 0.2713	0.32 (0.16, 0.47) <0.0001
*P* for trend	0.02 (-0.01, 0.04) 0.1779	0.02 (0.00, 0.03) 0.0049	-0.12 (-0.36, 0.13) 0.3551	7.86 (1.60, 14.11) 0.0139	1.10 (-0.42, 2.61) 0.1566	0.21 (0.11, 0.32) <0.0001
Model 3						
Continuous	-0.01 (-0.02, 0.01) 0.2078	0.01 (0.01, 0.02) 0.0004	-0.03 (-0.19, 0.13) 0.7357	3.68 (-0.42, 7.77) 0.0783	-0.52 (-1.51,0.46) 0.2997	0.15 (0.08, 0.22) <0.0001
Tertile 1	Ref.	Ref.	Ref.	Ref.	Ref.	Ref.
Tertile 2	0.03 (-0.00,0.06) 0.0697	0.01 (-0.00, 0.03) 0.1607	-0.35 (-0.71, 0.01) 0.0585	6.32 (-2.86, 15.50) 0.1771	-0.18 (-2.38,2.03) 0.8735	0.14 (-0.02, 0.29) 0.0814
Tertile 3	0.06 (0.03, 0.10) 0.0001	0.02 (0.00, 0.03) 0.0287	-0.35 (-0.71, 0.01) 0.0548	9.99 (0.90, 19.09) 0.0314	4.04 (1.86, 6.23) 0.0003	0.33 (0.18, 0.48) <0.0001
Tertile 4	0.02 (-0.01, 0.06) 0.1444	0.02 (0.01, 0.04) 0.0051	-0.26 (-0.63, 0.10) 0.1585	12.40 (3.14, 21.66) 0.0087	0.99 (-1.24, 3.22) 0.3834	0.30 (0.15, 0.46) 0.0001
*P* for trend	0.01 (-0.01, 0.03) 0.2906	0.01 (0.00, 0.03) 0.0071	-0.12 (-0.36, 0.13) 0.3604	8.03 (1.77, 14.30) 0.0120	0.89 (-0.62, 2.40) 0.2476	0.20 (0.10, 0.31) 0.0001

^a^Model 1: no covariates were adjusted. Model 2: adjusted for gender, age, and race. Model 3: adjusted for gender, age, race, education level, and marital status;

SIRI, Systemic Immune Inflammation Index;

TT3, Free Triiodothyronine;

FT4, Free Thyroxine;

TSH, Thyroid stimulating hormone;

TPOAb, Thyroid peroxidase antibodies;

TT3, Total Triiodothyronine;

TT4, Total Thyroxine;

CI, confidence interval.

**Figure 2:**
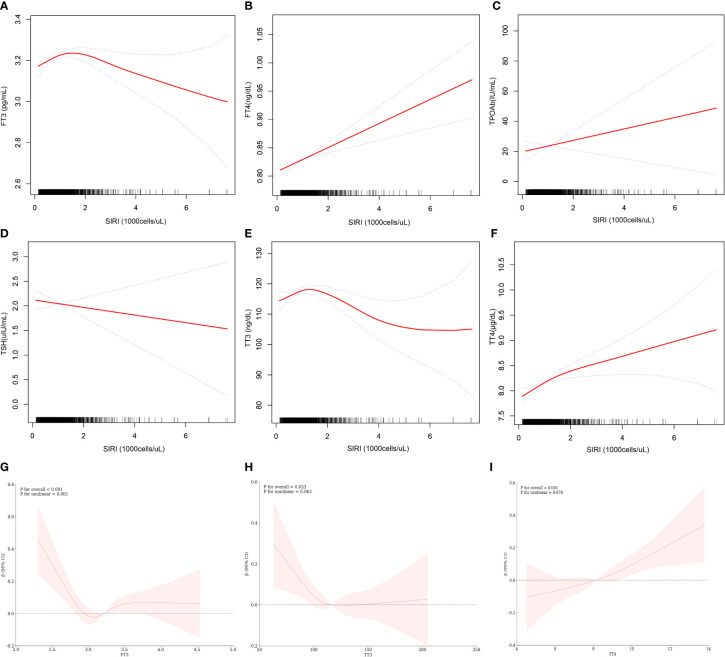
Relationship between SIRI and thyroid function. **(A–F)** is a curve-fit plot of SIRI versus thyroid function (FT3, FT4, TPOAb, TSH, TT3, and TT4). **(G–I)** is a restricted cubic spline plot of SIRI versus FT3, TT3, and TT4.

**Table 4** and **Figure 2** further elucidate the association between adjusted SIRI and thyroid function parameters. Utilizing a generalized additive model and restricted cubic spline (RCS) curves, which accounted for relevant confounding factors, a nonlinear association was discerned between SIRI and FT3, TT3, and TT4. Critical breakpoints (K) were determined for each hormone: 1.17 for FT3, 1.19 for TT3, and 1.18 for TT4. On the left side of these breakpoints, a positive correlation existed between SIRI and the hormones (FT3: *β* = 0.09, 95% CI = 0.04, 0.14, *P* for trend = 0.0003; TT3: *β* = -3.10, 95% CI = -4.47, -1.73, *P* for trend <0.0001; TT4: *β* = 0.26, 95% CI=0.05, 0.46, *P* for trend = 0.0138). Conversely, on the right side of the breakpoints, a significant negative correlation was observed between SIRI and FT3 and TT3, but not with TT4 (FT3: *β* = -0.04, 95% CI = -0.06, -0.02, *P* for trend = 0.0001; TT3: *β* = 5.58, 95% CI = 2.62, 8.55, *P* for trend = 0.0002; TT4: *β* = 0.05, 95% CI = -0.05, 0.14, *P* for trend = 0.3344). The log-likelihood ratio tests yielded *P* about <0.001, <0.001, and 0.106, respectively.

**Table 4:** Threshold effect analysis of the relationship between SIRI and thyroid function.

Outcome	FT3 (pg/mL)	FT4 (ng/dL)	TSH(uIU/mL)	TPOAb (IU/mL)	TT3 (ng/dL)	TT4(µg/dL)
Model I						
One linear effect	-0.01 (-0.03, 0.01) 0.2162	0.01 (0.01,0.02) <0.0001	0.01 (-0.15,0.17) 0.9175	2.68 (-1.31, 6.67) 0.1883	-0.91 (-1.91,0.09) 0.0743	0.10 (0.03, 0.17) 0.0046
Model II						
Breakpoint (k)	1.17	0.48	0.45	0.8	1.19	1.18
<K segment effect 1	0.09 (0.04,0.14) 0.0003	-0.06 (-0.18,0.06) 0.3453	1.63 (-1.76,5.01) 0.3466	27.91 (5.02, 50.79) 0.0169	5.58 (2.62,8.55) 0.0002	0.26 (0.05, 0.46) 0.0138
>K segment effect 2	-0.04 (-0.06, -0.02) 0.0001	0.01 (0.01, 0.02) <0.0001	-0.01 (-0.18, 0.15) 0.8763	0.02 (-4.62, 4.66) 0.9932	-3.10 (-4.47,-1.73) <0.0001	0.05 (-0.05, 0.14) 0.3344
Effect difference between 2 and 1	-0.13 (-0.19, -0.07) <0.0001	0.07 (-0.05, 0.20) 0.2461	-1.64 (-5.07, 1.79) 0.3486	-27.89 (-52.80, -2.97) 0.0283	-8.68 (-12.42,-4.95) <0.0001	-0.21 (-0.47, 0.05) 0.1066
Predicted value of equation at breakpoint	3.20 (3.18, 3.22)	0.81 (0.80, 0.82)	2.03 (1.85, 2.21)	24.77 (20.20, 29.34)	116.65 (115.35,117.94)	8.00 (7.91, 8.09)
LRT test	<0.001	0.246	0.348	0.028	<0.001	0.106

For exposure: Systemic inflammatory response index.

Model I, linear analysis; Model II, nonlinear analysis.

LRT test, logarithmic likelihood ratio test. (P < 0.05 denotes that Model II significantly differs from Model I, revealing a nonlinear relationship);

TT3, Free Triiodothyronine;

FT4, Free Thyroxine;

TSH, Thyroid stimulating hormone;

TPOAb, Thyroid peroxidase antibodies;

TT3, Total Triiodothyronine;

TT4, Total Thyroxine;

CI, confidence interval.

### Subgroup analysis of SIRI and thyroid function

**Figure 3:**
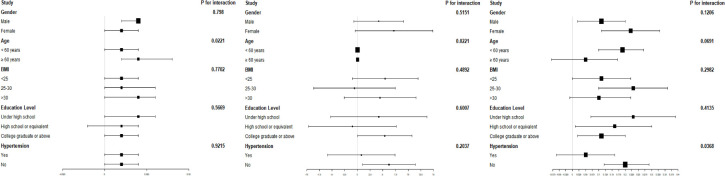
Subgroup analysis of SIRI and thyroid function.

**Figure 3** presents subgroup analyses examining the relationship between the SIRI and thyroid function. To ascertain if SIRI’s association with thyroid function is consistent across the general population and to identify distinct subgroups, we conducted analyses stratified by age, gender, BMI, and hypertension, along with interaction tests. Our findings reveal significant interactions of SIRI with FT4 in different age groups, with TT4 in the context of hypertension, and with TPOAb across age groups (all *P* for trend <0.05). However, no notable differences were observed in terms of gender and BMI. Thus, our study indicates that SIRI’s correlation with thyroid function varies with hypertension status and may be relevant for individuals without hypertension. Importantly, our interaction tests did not reveal any significant differences associated with gender and BMI, indicating that these factors do not influence the relationship between SIRI and thyroid function.

## Discussion

The objective of this study was to evaluate the association between the SIRI and thyroid function in a population of US adults. In our cross-sectional analysis of 1,641 participants, we discovered a significant positive correlation between SIRI and both FT4) and TTT4. However, no significant correlation was found between SIRI and TSH. Adjustments for all covariates revealed elevated levels of FT4, TT4, and TPOAb in the highest quartile of SIRI (Tertile 4) compared to the lowest (Tertile 1). Additionally, we employed Generalised Additive Models and Restricted Cubic Splines to further delineate the SIRI-thyroid function relationship. Our findings indicated a nonlinear association between SIRI and FT3, TT3, and TT4. Notably, the nature of the relationship varied across the breakpoint; SIRI was positively correlated with FT3, TT3, and TT4 on the left side of the breakpoint, whereas on the right side, it was negatively correlated with FT3 and TT3. Still, the correlation with TT4 was not statistically significant. Finally, the interaction term analysis suggested that gender and BMI did not significantly influence these associations.

This study is the first to investigate the correlation between SIRI and thyroid function risk. Previous research has established connections between thyroid function and various clinicopathological factors. Chen S et al. observed a negative relationship between the Healthy Eating Index (HEI) and levels of FT3 and TT3. Additionally, they found a strong association between the Dietary Inflammatory Index (DII) with increased FT3 and TT4 levels, suggesting DII’s utility in evaluating dietary impacts on inflammatory potential (6). Anti-inflammatory diets, such as DASH and Mediterranean, have been noted to lower systemic inflammation levels (7, 8). Research indicates that gut flora and dietary fiber modulate inflammatory states via inter-organ signaling, thus impacting pathophysiological aspects of obesity, diabetes, and dyslipidemia (9, 10). Leonardo et al. demonstrated that pro-inflammatory diets can lead to intestinal imbalance, bacterial overgrowth, increased intestinal permeability, and oxidative stress. This was evidenced by examining the faces of HT patients, suggesting that such an inflammatory response could accelerate HT development and support the notion of a thyroid-gut axis (6). GlycA, a glycoprotein biomarker, indicates systemic inflammation by measuring acute phase reactants and various glycosylated proteins. Research by Nilay Yukse et al. found that patients with hypothyroidism, regardless of treatment status, exhibited elevated GlycA levels, indicative of low-grade systemic inflammation. Similarly, Nizamutdinova IT identified a strong link between hyperthyroidism, inflammatory responses, apoptosis, and activation of hypertrophy-related proteins. Hyperthyroidism triggers NF-kB activation, escalating NO, lipid peroxidation, and free radical production. This results in a marked increase in TNF-a, IL-1b, IL-4, IL-6, and IL-10 levels (5, 11). A meta-analysis further confirmed significantly higher serum CRP levels in hypothyroid patients (12), corroborating previous findings (13). It has been demonstrated that obesity-induced adipose tissue expansion provides many internal signals that may trigger an inflammatory response (e.g. adipocyte death, hypoxia, and mechanical stress). A cohort study verified that overweight and obesity are linked to elevated free T3 levels. Altered thyroid function in these patients increases energy expenditure, aiding in weight loss and preventing further weight gain. Consequently, TSH and FT3 normalization post-weight loss may contribute to the challenges in maintaining weight loss (14). Given the association between inflammation, abnormal thyroid function, and the reliability of SIRI as an inflammation indicator, a positive correlation between SIRI and thyroid function appears likely.

Prior epidemiological studies indicate that hypertension, obesity, and gender significantly impact thyroid function. Ladan Mehran et al. found that hypertension diminishes TH sensitivity (15). Research has also linked obesity to a heightened risk of hypothyroidism (16), possibly due to obesity’s nature as a chronic, low-grade inflammatory state. In this state, inflammatory markers such as interleukin-1 (IL-1), IL-6, and tumor necrosis factor-alpha (TNF-α), produced by overloaded adipose tissue, increase (17). Estrogen, a key growth factor for thyroid cells, promotes growth through genomic and non-genomic pathways via the membrane-bound estrogen receptor. This receptor is associated with the MAPK and PI3K tyrosine kinase signaling pathways (18), potentially explaining the higher prevalence of thyroid diseases in women compared to men. According to our subgroup analysis, there was a significant SIRI with FT4 in age (*P* for interaction = 0.0221), SIRI with TT4 in hypertension (*P* for interaction = 0.368), and SIRI with TPOAb in age (*P* for interaction = 0.0221). These findings suggest that the relationship between the SIRI and thyroid function is influenced by hypertension. Hyperthyroidism has been linked to increased cardiovascular disease risk and endothelial dysfunction (19). It may also lead to secondary systolic hypertension through elevated cardiac output and increased renin, angiotensin, and aldosterone levels. Notably, a significant positive correlation between SIRI and FT4, TT4, and TPOAb persists in nonhypertensive subgroups, underscoring its relevance in this population.

The underlying mechanism driving the positive correlation between the SIRI and thyroid hormones FT4, TT4, and TPOAb remains elusive. However, existing research sheds light on this association. It’s established that thyroid diseases prompt alterations in TH and related regulatory hormones. For instance, hyperthyroidism results from an excessive release of TH (T3 and T4), leading to protein, lipid, and DNA damage due to stress, increased basal metabolic rate, and oxygen consumption in body tissues (20). HT stems from an autoimmune attack on thyroid tissue, characterized by lymphocytic infiltration, fibrosis, and interstitial atrophy. RET/PTC rearrangements, implicated in HT, activate pathways that induce both HT and the expression of genes like CXCL5 and CXC chemokine receptor 2 (CXCR2), linked to inflammation and tumor invasion (21). Furthermore, Galdiero (22) et al. observed that in thyroid cancer patients, neutrophils upregulate CD 11b and CD 66b, correlating tumor size. Collectively, these findings strongly suggest a link between thyroid dysfunction and inflammation. SIRI emerges as a comprehensive marker of the body’s inflammatory and immune response, surpassing traditional parameters like the neutrophil-to-lymphocyte ratio (NLR) and platelet-to-lymphocyte ratio (PLR) in predictive capability. This extends to tumor prognosis, cardiovascular disease, chronic obstructive pulmonary disease, and COVID-19. Yuxiu Yang et al.’s study with 1527 patients with moderate coronary artery stenosis exemplifies SIRI’s superior performance over conventional cardiovascular risk models (23). Multiple studies corroborate these findings, highlighting SIRI’s reliability and potential applications. In our study, accounting for confounding factors, we identified a positive correlation between FT4, TT4, and TPOAb, indicating SIRI’s role in the pathogenesis and progression of thyroid disorders. Thus, monitoring SIRI levels could provide essential insights for therapeutic decision-making and tracking disease progression in thyroid diseases. This study paves the way for further investigation and underscores the role of inflammation in thyroid disorders.

The robust sample size and thorough adjustment for covariates enhance the credibility and representativeness of our study. Nevertheless, it is not without limitations. The cross-sectional design precludes causal inference, necessitating future prospective studies with larger cohorts to clarify causality. Our reliance on data from a public dataset, NHANES, meant we could not modify the study design. While we accounted for several covariates, potential confounders, such as medication use (e.g., steroids), could still have influenced the outcomes. Unfortunately, NHANES did not collect data on these variables, restricting our ability to incorporate them into our analysis.

Furthermore, as NHANES is a U.S.-based study, our evaluation was confined to the association between SIRI and thyroid function in the American adult population. The limitation to a single-country sample with restricted ethnic diversity hinders the generalizability of our findings. In summary, while our study indicates a correlation between elevated SIRI levels and increased FT4, TT4, and TPOAb levels, and a nonlinear relationship among FT3, TT3, and TT4, further research is essential for validation.

## Ethics Approval

The studies involving human participants were reviewed and approved by the ethics review board of the National Center for Health Statistics.

## Informed consent

The patients/participants provided their written informed consent to participate in this study.

## Data availability statement

The original contributions presented in the study are included in the article/supplementary material. Further inquiries can be directed to the corresponding author.

## Author contributions

YZ: Writing – original draft, Data curation, Investigation. BW: Writing – review & editing, Conceptualization, Methodology. WH: Writing – review & editing, Supervision, Validation. BY: Writing – review & editing, Formal Analysis, Investigation. JC: Writing – review & editing, Validation. FA: Writing – review & editing, Validation.
